# Longitudinal association of grip strength with cardiovascular and all-cause mortality in older urban Lithuanian population

**DOI:** 10.1186/s12889-024-18506-w

**Published:** 2024-04-15

**Authors:** Lolita Sileikiene, Abdonas Tamosiunas, Dalia Luksiene, Ricardas Radisauskas, Daina Kranciukaite-Butylkiniene, Martin Bobak

**Affiliations:** 1 Institute of Cardiology, Medical Academy, Lithuanian University of Health Sciences, Kaunas, Lithuania; 2 Institute of Epidemiology and Health Care, University College London, London, UK

**Keywords:** Grip strength, All-cause mortality, CVD mortality risk, Longitudinal study

## Abstract

**Background:**

Ageing populations experience greater risks associated with health and survival. It increases the relevance of identifying variables associated with mortality. Grip strength (GS) has been identified as an important biomarker for all cause and cardiovascular mortality, however, its prognostic value has not been studied in Lithuania. The aim of the present study is to evaluate the relationship of GS to vital status in a representative sample of the Lithuanian 45–72-year-old urban population during the period of 12 years of follow-up and to explore associations of GS with all-cause mortality and mortality from cardiovascular diseases (CVD).

**Methods:**

Within the framework of the international study Health, Alcohol and Psychosocial Factors in Eastern Europe (HAPIEE) 7,115 men and women 45–72 years of age were examined in the baseline survey (2006 to 2008). Data from the Official Lithuanian Mortality Register were used to evaluate CVD and all-cause mortality from follow-up till 2020. Cox proportional hazards regression was used, and four models for all-cause and CVD mortality were assessed.

**Results:**

The mean GS was significantly higher among survivors’ men and women as compared to individuals deceased from CVD and other causes of death. In survivor men and women groups, minimal values of GS in all terciles were higher as compared to all three deceased groups. In both men and women groups, the lowest GS (1st tercile) was associated with a significantly higher risk of all-cause and CVD mortality as compared to the highest levels of GS (3rd tercile) in three Cox regression models. In both men and women were found to have a 1.34- and 1.35-fold higher risk of all-cause mortality, respectively, at lower GS, but no significant difference in the risk of CVD mortality. When GS was treated in all models as decrement per 1 kg and decrement per 1 SD, in both men and women, the risk of all-cause mortality significantly increased with decreasing of GS.

**Conclusions:**

The mean GS was significantly higher among survivors’ men and women as compared to deceased from CVD and other causes of death. Risk of all-cause mortality significantly increased with decreasing of GS.

## Introduction

Process of demographic ageing has affected population health, older people having expectations to spend their later life years in relatively good health, remaining active participants in society. Conventional indicators of population ageing that are based on chronological age (years since birth), with a fixed threshold of “old age” at age 65, show that populations are becoming older in all regions of the world [1]. It is projected that in 2050 people aged 65 years or more will make a considerable part of the population in Europe [2].

Active aging [3, 4] is critical to extend healthy life expectancy and quality of life. A grave change associated with human aging is a progressive decline in skeletal muscle mass, a downward spiral that may lead to decreased strength and functionality [5]. Objective measures of physical capability are predictors of all-cause mortality in older community-dwelling populations; such measures may provide useful tools for identifying older people at higher risk of death [6, 7]. One way of measuring muscle function is to measure GS. GS measured by dynamometry is well established as an indicator of muscle status, particularly among older adults [8]. GS has been introduced as a biomarker [9] of aging, as well as a biomarker of current and future medical status, and a strong predictor of mortality [10].

GS has been proclaimed as a biomarker for older adult populations, both, as an indicator of current health status, a biomarker of future outcomes, as well as a predictor of future function and changes in function over time [11].

Hand-grip dynamometry, the measurement of GS has been widely adopted as a singular indicator of overall strength [11]. Studies have confirmed the value of GS as a predictor of physical functioning, morbidity, and disability in old age, and mortality, as well as an indicator of health outcomes in healthcare, such as hospital length of stay [8, 12, 13]. An inverse association between muscle strength and cardiovascular health has been reported, and poor cardiovascular outcomes have been demonstrated in initially healthy individuals of all age groups, and in those with existing disease [14]. Investigations have shown that baseline higher GS was associated with lower risk of all-cause and cause specific mortality and incidence [15, 16].

Low GS is a proclaimed risk factor for mortality [8]. While a systematic review with dose-response meta-analysis provides information about strong evidence for an association between lower GS with higher all-cause, cancer, and cardiovascular mortality risk [17], evidence concerning the prognostic value of GS is sometimes conflicting. However, GS may be considered a useful prognostic tool for CVD events in the population [18]. Recent data from a systematic review and meta-regression analysis concluded that protocols used to assess GS in mortality studies were incomplete and highly heterogeneous. GS values were found to be higher when studies controlled fewer GS measurement protocol variables [12].

To the best of our knowledge, our paper is the first to investigate the association between GS and all-cause, as well as CVD mortality, in Lithuania, the country where the share of aging people is growing fast, but also in other Baltic countries such as Estonia and Latvia. In Lithuania, morbidity and mortality from CVD are almost twice as high as the EU average [19]. Therefore, it is very important to assess the prognostic significance of factors other than traditional risk factors for morbidity and mortality from major chronic diseases. GS is one such factor that is reasonably easily and accurately measured in various populations. Our research results could lead to insights about the timing of muscle mass and GS decline in older adults and the potential role of the former state transitions in their lives in relation to mortality.

The aim of the present study is to evaluate the relation of GS to vital status in a representative sample of the Lithuanian 45–72-year-old urban population during the period from 2006 to 2020 and to explore associations between GS and mortality from CVD and other causes.

## Methods

### Study sample

Initial data were collected within the framework of the international HAPIEE (Health, Alcohol and Psychosocial Factors in Eastern Europe) study in 2006–2008 [20]. Data from the survey in a population-based urban cohort study conducted in Kaunas (Lithuania) is presented. Baseline data collection of men and women aged 45–72 years has been executed between 2006 and 2008. It recruited 7,115 participants from a study sample of 10,980 individuals, randomly selected from the National Population Register, the response rate in the baseline survey was 64.8%. Data from the Official Lithuanian Mortality Register were used for evaluation of CVD and all-cause mortality during the period of follow-up till 2020 (including). Causes of death were coded by the International Classification of Diseases (ICD) (version 10): deaths of CVD included codes I00-I99, and all causes of death included codes A00-Z99. In this study, 168 participants were excluded from the analysis due to incomplete information about GS.

The HAPIEE study has received ethical approval from the Kaunas Regional Biomedical Research Ethics Committee (11 January 2005; No. 05/09) and by the Ethics Committee at the University College London. All participants signed the form of informed consent to participate in the survey and were allowed to use their medical documents during follow-up.

### Variables

The variables were determined at the baseline survey using the questionnaire by the HAPIEE study protocol [20]. The questionnaire included questions regarding the respondent’s age, sociodemographic factors (education, marital status, social activity, employment status), psychological factors (depressive symptoms, cognitive function, psychological well-being, self-rated quality of life), lifestyle variables (smoking habits, alcohol consumption frequency, physical activity),, existing illness at baseline, etc. The cut-offs of the variables were used in other scientific analyses, as well [21–23].

### Grip strength

Measurements of GS have been done in the morning hours and the maximum GS of each hand has been measured [20]. GS (in kg) measurements were performed with a Smedley spring-type handgrip dynamometer (Cat. No: 281,128, 100 kg). Subjects performed the measurements while standing with their arms bent at a 90-degree angle. Study participants were asked to squeeze the dynamometer as hard as they could with their arm at their side and elbow bent at 90 degrees while standing. Four measurements were taken with both hands in succession, with 5 s breaks between measurements. The GS size was calculated from the average of the two measurements of the dominant arm.

The respondents were categorized into three groups according to their GS (in kg) and of each tercile min and max values in men and women groups are presented in Table 1.

**Table 1:** Baseline characteristics of the Kaunas HAPIEE study sample according to grip strength terciles and sex

			Grip	Strength	terciles			Variables
Variables		MEN			WOMEN			
	1st tercile	2nd tercile	3rd tercile	1st tercile	2nd tercile	3rd tercile	MEN *N* = 3,139	WOMEN *N* = 3,733
**Grip strength**, kg, median/mean ± SD	39.0	47.0	55.0	23.0	29.0	34.0	48.1 ± 8.67	29.2 ± 6.09 *
**SBP** mm Hg, median/mean ± SD	145.0	142.7	141.0 ^**c**^	135.0	133.0 ^**d**^	131.0 ^**c**^	144.6 ± 21.14	134.5 ± 20.94 *
**BMI** kg/m^2^, median/mean ± SD	27.3	27.6 ^**d**^	28.6 ^**c**^	29.7 ± 5.82	29.4 ± 5.66	29.4 ± 5.84	28.3 ± 4.60	29.5 ± 5.77 *
**Total Chol** mmol/L, median/mean ± SD	5.69 ± 1.11	5.84 ± 1.11 ^**a**^	5.86 ± 1.07 ^**a**^	6.03	6.02	5.93	5.80 ± 1.10	6.06 ± 1.12 *
**Tg** mmol/L, median	1.19	1.23 ^**d**^	1.31 ^**c**^	1.27	1.23	1.20 ^**c**^	1.25	1.22
**Fasting glucose** mmol/L, median	5.60	5.60	5.60	5.70	5.60	5.60 ^**a**^	5.60	5. 60.
**Smoking status, %**								
Current	33.8	35.6	36.8	10.2	13.6 ^**e**^	16.1^**e**^	35.6	13.6 ^**#**^
Former	27.9	30.2	28.8	5.3	8.2 ^**e**^	10.9 ^**e**^	29.0	8.4 ^**#**^
Never	38.3	34.2	34.4	84.5	78.2 ^**e, f**^	73.1 ^**e**^	35.5	78.1 ^**#**^
**Alcohol consumption, %**								
Every day or almost every day	6.7	6.3	6.2	1.4	0.9	0.9	6.4	1.1 ^**#**^
2–4 times per week	14.8	17.2	17.8	3.4	2.7	3.1	16.8	3.1 ^**#**^
Once a week	13.5	16.6	19.7 ^**e**^	5.7	8.4 ^**e**^	9.6 ^**e**^	17.0	8.1^**#**^
1–3 times per month	33.3	35.0	34.4	28.7	33.1 ^**f**^	38.5 ^**e**^	34.3	33.8
Less than once a month	24.9	20.2 ^**e**^	17.2 ^a^	50.3	48.5 ^**f**^	44.1 ^**e**^	20.3	47.4 ^**#**^
Never	6.8	4.7	4.6	10.4	6.4 ^**e, f**^	3.6 ^**e**^	5.3	6.5 ^**#**^
**Physically active**, **%**	67.3	64.8	67.8	77.0	81.5 ^**e**^	83.9 ^**e**^	66.7	81.1 ^#^
**Sociodemographic factors**:								
**Age, years**, median/mean ± SD	65.0	59.0 ^**c, d**^	53.0 ^**c**^	64.0	58.0 ^**c, d**^	53.0 ^**c**^	57.4 ± 7.96	57.2 ± 7.89
**Marital status, %**								
Married	79.0	83.3	86.6 ^**e**^	52.5	58.4 ^**e, f**^	63.9 ^**e**^	83.4	58.9 ^**#**^
Single	2.3	3.0 ^**f**^	1.1	5.5	6.4	7.0	2.0	6.3 ^**#**^
Living unmarried	1.5	2.0	1.9	0.5	0.9	1.7 ^**e**^	1.8	1.1 ^**#**^
Divorced	10.2	8.7	7.5	16.9	17.2	17.2	8.6	17.1 ^**#**^
Widowed	7.1	3.0 ^**e**^	2.9 ^**e**^	24.7	17.1 ^**e, f**^	10.2 ^**e**^	4.1	16.6 ^**#**^
**Education, %**								
Primary	11.0	4.1 ^**e, f**^	1.1 ^**e**^	9.4	4.2 ^**e, f**^	1.5 ^**e**^	4.8	4.7
Vocational	13.9	9.7 ^**e, f**^	5.0 ^**e**^	9.9	6.9 ^**e, f**^	3.9 ^**e**^	9.0	6.6 ^**#**^
Secondary	31.6	35.3	32.4	29.1	25.4	24.0 ^**e**^	33.0	25.9 ^**#**^
College	16.8	17.9	21.7 ^**e**^	23.9	29.0 ^**e**^	31.5 ^**e**^	19.1	28.5 ^**#**^
University	26.7	33.1 ^**e, f**^	39.8 ^**e**^	27.7	34.6 ^**e**^	39.1 ^**e**^	34.0	34.2
**Employment status, %**								
Employed	27.5	53.2 ^**e, f**^	73.4 ^**e**^	23.3	47.0 ^**e, f**^	66.9 ^**e**^	53.5	47.4 ^**#**^
Employed-retired	19.2	15.6 ^**f**^	8.6 ^**f**^	12.3	12.1 ^**f**^	8.2 ^**e**^	13.7	10.5 ^**#**^
Employed-disabled	4.5	4.7	4.7	4.5	4.6	4.1	4.8	4.6
Unemployed	3.3	2.7	3.9	3.5	4.4	4.1	3.3	4.1
Retired	30.1	14.9 ^**e, f**^	4.6 ^**e**^	40.3	24.9 ^**e, f**^	11.3 ^**e**^	15.3	24.1^**#**^
Disabled	15.4	8.8 ^**e, f**^	4.8 ^**e**^	16.2	6.9 ^**e**^	5.3 ^**e**^	9.4	9.2
**Social activity, %**								
Low	43.0	30.2 ^**e, f**^	20.7 ^**e**^	34.8	23.3 ^**e, f**^	18.9 ^**e**^	30.0	24.9 ^**#**^
Medium	35.8	35.2	32.9	38.4	40.9	37.8	34.4	39.0 ^**#**^
High	21.2	34.5 ^**e, f**^	46.5 ^**e**^	26.8	35.9 ^**e, f**^	43.2 ^**e**^	35.6	36.1
**IHD, %**	21.3	14.8 ^**e, f**^	13.2 ^**e**^	21.4	17.2 ^**e, f**^	14.8 ^**e**^	15.2	17.2
**Previous stroke, %**	6.1	3.6 ^**e, f**^	1.7 ^**e**^	5.7	3.2 ^**e, f**^	0.8 ^**e**^	3.9	3.1
**Normal cognitive function, %**	75.4	80.8 ^**e**^	81.9 ^**e**^	85.0	88.7 ^**e**^	91.1 ^**e**^	79.5	88.4 ^**#**^
**Depressive symptoms, %**	20.7	14.6 ^**e**^	12.5 ^**e**^	37.8	26.4 ^**e, f**^	22.0 ^**e**^	15.7	28.6 ^**#**^
**Higher psychological well-being, %**	46.5	53.4 ^**e**^	58.5 ^**e**^	46.3	57.5 ^**e, f**^	65.2 ^**e**^	53.1	56.7
**Self-rated quality of life, %**								
Very poor and poor	4.1	4.1 ^**f**^	1.8 ^**f**^	7.9	3.8 ^**e, f**^	1.8 ^**e**^	3.1	4.2 ^**#**^
Average	55.1	46.0 ^**e, f**^	39.3 ^**e**^	55.3	47.9 ^**e, f**^	40.3 ^**e**^	45.8	47.1
Good	39.4	48.4 ^**e, f**^	56.3 ^**e**^	36.0	46.6 ^**e, f**^	55.8 ^**e**^	49.0	47.1
Very good	1.4	1.6	2.7	0.8	1.7 ^**f**^	2.0 ^**f**^	2.0	1.6

BMI: Body Mass Index; Chol: cholesterol; IHD: Ischemic Heart Disease; SD: Standard Deviation; SBP: Systolic Blood Pressure; Tg: Triglycerides

^**a**^*p* < 0.05 as compared to 1st tercile; ^**b**^*p* < 0.05 as compared to 3rd tercile (ANOVA test); *p* values have been adjusted by the Bonferroni correction for multiple comparisons

^**c**^*p* < 0.05 as compared to 1st tercile; ^**d**^*p* < 0.05 as compared to 3rd tercile (Kruskal-Wallis test); *p* values have been adjusted by the Bonferroni correction for multiple comparisons

^**e**^*p* < 0.05 as compared to 1st tercile; ^**f**^*p* < 0.05 as compared to 3rd tercile (chi^2^ and Z-test); *p* values have been adjusted by the Bonferroni correction for multiple comparisons

*p < 0.05 as compared to men (T-test)

^**#**^*p* < 0.05 as compared to men (Z-test)

### Sociodemographic factors: age, marital status, education, social activity, employment status

The study questionnaire included questions regarding the respondent’s age, marital status, education, social activity, and employment status [21, 23].

Baseline sample (data collected between 2006 and 2008) consisted of 10,980 Kaunas men and women *aged* 45–72 years, selected from the National Population Register, stratified by sex and age (response rate 64.8%) [21, 23]. Five categories of *marital status* (married, cohabiting, single, widowed, and divorced) were listed in the questionnaire. *Education* was classified into five education levels: primary, vocational, secondary, college, and university. *The social activity* of study participants was *evaluated* by statements about participating in clubs, going to church, theatres, sports clubs, and restaurants. Respondents were categorized into three groups - low, moderate, and high social activity. *Employment* status was defined by classifying the participants into six categories: employed, employed-retired, employed-disabled, unemployed, retired, and disabled.

### Psychological factors: depressive symptoms, cognitive function, psychological well-being, self-rated health, and quality of life

#### Depressive symptoms

Depressive symptoms were measured using the 10-item Centre for Epidemiologic Studies Depression Scale (CES-D 10) [24]. The respondents were interviewed by specially trained personnel who filled in the questionnaires. The subjects were asked to evaluate the presence of ten depressive symptoms during the past week on a two-point scale: “Yes” or “No”. Each symptom was scored 0 (“No”) or 1 (“Yes”), resulting in a total score of 0–10. The subjects who scored 4 or more CES-D 10 scores were classified as having depressive symptoms.

### Cognitive function

Assessment of cognitive function was carried out in a separate consulting room, in the morning hours, by specially trained staff [20]. Tests of cognitive function have been taken from the English Longitudinal Study of Ageing (ELSA) and Consortium to Establish a Registry for Alzheimer’s Disease (CERAD) study [25, 26]. Cognitive function tests involved three immediate and one delayed recall of 10 words, animal naming in 1 min, and letter cancellation in 1 min [20].

*Immediate and delayed verbal memory* was assessed using a 10-word learning test. This involved 10 common two-to-four-syllable nouns being presented aurally by a tape recorder at the rate of one word every 2 s. The participants were then asked to recall as many words as possible immediately and they had two minutes to do so. The test was repeated three times using the same procedure. The maximum score of any trial is 10 (range: 0–10). The cumulative total maximum score over all three learning trials was 30 (range: 0–30). The participants were also asked to recall as many words as possible again after an approximately five-minute delay during which they completed other cognitive function tests. The maximum score of delayed verbal memory is 10 (range: 0–10). *Semantic verbal fluency* was examined by asking the participants to name as many animals as possible within 1 min. *Speed and concentration* were tested by asking the participants to cross out as many target letters (‘P’ and ‘W’) as possible within 1 min, using a sheet with random letters of the alphabet set out in rows and columns. Numerical ability was assessed using four questions involving simple calculations based on everyday situations. The number of correct responses comprised the numeracy score (in the range of 0 to 4).

#### Psychological well-being (PWB)

PWB was evaluated by a Control Autonomy Self-realization and Pleasure (CASP-12) questionnaire [27], which is composed of 12 statements. Participants indicated how often (often, sometimes, not often, never) each statement applied to them. The total score ranges from 12 to 48, where a higher score represents a higher PWB. The internal consistency of the scale was good (Cronbach’s *α* = 0.74). The scale was dichotomized to produce two groups of PWB, one consisting of participants with high PWB and the other of participants with lower PWB. Participants were classified as having a higher PWB if the CASP-12 score was higher or equal to the median: ≥ 40 in men and ≥ 38 in women.

#### Self-rated health and quality of life

The study questionnaire included questions regarding the respondent’s self-rated health and quality of life: “Over the last 12 months, would you say your health has been? and “How would you rate your quality of life?” Self-rated health and quality of life have been evaluated according to the answers of the study participants to these questions. According to their answers, the respondents were categorized into the following groups of self-rated health and quality of life: very good, good, average, poor, and very poor.

### Lifestyle variables

Lifestyle factors were evaluated using standard questionnaire and some anthropometric measurements were performed.

*Smoking status* was classified as never smoking, former smoking and current smoking. Current smokers were individuals who regularly smoked at least 1 cigarette per day.

*Alcohol drinking frequency* was categorized as never, less than 1 time per month, 1 to 3 times per month, once per week, 2 to 4 times per week, every day. Respondents reported the quantity of spirits, beer, and wine usually consumed per week.

To assess the *physical activity* of the participants in their leisure time, 5 questions were asked. Physical activity was determined by the mean length of time spent per week during leisure time in autumn-winter and spring-summer seasons for walking, moderate and hard work, such as gardening, maintenance of the house, and other physical activities, such as engage in sports, games, or hiking. The participants were categorized as physically active (physical activity of 10 h or more per week) and inactive (physical activity of less than 10 h per week).

### Other covariates

Covariate variables were determined at baseline survey using measurements of height, weight, blood pressure, and biochemical analyses (total cholesterol, triglyceride (Tg), and fasting glucose (FG)).

*The body weight and height* were measured with a calibrated medical scale. Body mass index (BMI) was calculated as the weight in kilograms divided by the height in meters squared (kg/m^2^) [22]. We divided study participants into groups: group with normal weight (BMI 18.5–24.99 kg/m²), overweight (BMI 25.0–29.99 kg/m²), and obesity (BMI ⩾30.0 kg/m²). Insufficient weight was defined as BMI < 18.5 kg/m².

*Blood pressure* was measured three times, with a two-minute interval between measurements, using an Omron M5-I digital blood pressure monitor, prior to blood pressure measurement participants were asked to sit quietly for 5 min [20]. The mean of three systolic (SBP) and diastolic blood pressure (DBP) was used [23]. Arterial hypertension was defined as systolic blood pressure ≥ 140 mm Hg and/or diastolic blood pressure ≥ 90 mm Hg, or normal blood pressure (< 140/90 mm Hg) if the person had taken antihypertensive drugs within the last two weeks [23].

Biochemical analyses were conducted for participants on an empty stomach; lipid concentrations in serum were measured, using a conventional enzymatic method; serum samples were analysed in the WHO Regional Lipid Reference Centre, Institute of Clinical and Experimental Medicine, Prague (Czech Republic). Concentration of glucose in capillary blood was determined by an individual glucometer “Glucotrend” [28].

*Coronary heart disease* (CHD) was determined using the following criteria: (1) a documented history of myocardial infarction (MI) and/or ischemic changes on electrocardiogram (ECG) coded by Minnesota codes (MC) 1–1 or 1–2 [29]; (2) angina pectoris was defined by G. Rose’s questionnaire (without a history of MI and/or MC 1–1 or 1–2) [30]; (3) ischemic changes on ECG coded by MC 1–3, 4 − 1, 4 − 2, 4 − 3, 5 − 1, 5 − 2, 5 − 3, 6 − 1, 6 − 2, 7 − 1, or 8 − 3 (without MI and/or MC 1–1, 1–2 and without angina pectoris). The previous stroke was determined according to a documented history of stroke.

CVD included CHD and/or stroke which were determined at baseline survey. The covariates and their classification are described in detail in our previous papers [31, 32].

### Statistical analysis

The analysis was performed for men and women (aged 45–72 years at baseline survey) separately. All continuous variables included in analysis were tested for normality (by skewness and kurtosis values) and homogeneity of variance (by Levene’s test). Descriptive characteristics (prevalence rates, medians, means, and standard deviations were calculated for variables in groups for GS terciles separately for men and women. The differences in means of variables between the sex groups and between the GS groups were assessed using T-test and ANOVA test for variables that were normally distributed and met the conditions of homogeneity of variance. For variables that did not accept the assumptions of normality and homogeneity of variance, nonparametric tests (Mann-Whitney and Kruskal Wallis) were performed to evaluate the differences between the sex groups and between the GS groups. A chi-squared test and z-test were used to assess the differences in categorical variables. Significance values have been adjusted by the Bonferroni correction for multiple comparisons; *p* < 0.05 values were considered statistically significant.

Hazard ratios (HR) and 95% confidence intervals (CI) were estimated by the Cox proportional hazards regression for all-cause and CVD mortality. Four models were assessed. Model 0: not adjusted. Model 1: adjusted for baseline age. Model 2: adjusted for all variables in Model 1 plus sociodemographic factors (education, marital status, social activity, and occupation). Model 3: adjusted for all variables in Model 2 plus psychological variables (depressive symptoms, cognitive functions, psychological well-being, and self-rated quality of life), lifestyle variables (smoking habits, alcohol consumption frequency, and physical activity), biologic variables (systolic blood pressure, total cholesterol, triglycerides, fasting glucose, and body mass index), and existing illness at baseline (diabetes, ischemic heart disease, and stroke).

For data analysis, the IBM SPSS Statistics (Version 27.0) (IBM Corp. Released 2020. IBM SPSS Statistics for Windows, Version 27.0. Armonk, NY, USA) software package was used.

## Results

During the 12-year follow-up period (for men 11.8 ± 3.54 years; for women 12.8 ± 2.52 years), 1,503 participants in the original study (*n* = 5,369) died; CVD was the main cause of death in 432 men and 266 women deceased. Table 2 summarizes baseline demographic and health-related factors, some chronic diseases of the HAPIEE study participants, stratified by terciles of GS and sex. The mean GS was 48.1 kg in men and 29.1 in women (*p* < 0.05). In the 3rd tercile of GS median age was lower both, in men and in women. In men, medians of BMI and Tg and mean of total Chol were significantly higher in the 3rd tercile of GS as compared to the 1st tercile. In women, the medians of SBP and FG were lower in the highest tercile of GS as compared to the lowest (*p* < 0.05). In both groups of men and women proportions of married individuals, individuals with university education, high level of social activity, higher level of psychological well-being, normal cognitive function, and proportion of self-rated quality of life as “good” were significantly higher in the 3rd tercile of GS as compared to the 1st tercile. On the opposite, a lower proportion of disabled individuals, individuals with depressive symptoms, the prevalence of IHD, previous stroke, and individuals who rated their quality of life as “poor” and “very poor” were significantly lower, both in men and women, in the highest tercile of GS as compared to the lowest tercile.

**Table 2:** Grip strength (kg) means (SD), frequencies and their confidence intervals (CI) according to vital status of 45–72-year participants*

Grip strength	All participants		Vital	status	
	at baseline	Alive	Dead from all causes of death	Dead from CVD	Dead from other than CVD causes of death
MEN	*N* = 3,139	*N* = 2,208	*N* = 931	*N* = 432	*N* = 499
Mean ± SD	48.1 ± 8.67	49.3 ± 8.30	44.3 ± 8.72 ^**a**^	44.1 ± 9.22 ^**b**^	44.5 ± 8.30 ^**b**^
	**min-max**	**%, CI**	**%, CI**	**%, CI**	**%, CI**
1st tercile	2.5–43.0	58.5 (55.56–61.3)	41.5 (38.7–44.4)	19.5 (17.3–21.9)	22.0 (19.7–24.5)
2nd tercile	43.5–50.5	75.8 (73.3–78.2)	24.2 (21.8–26.7)	10.6 (8.9–12.5)	13.6 (11.7–15.6)
3rd tercile	51.0–77.0	84.4 (82.4–86.2)	15.6 (13.8–17.6)	7.1 (5.9–8.5)	8.5 (7.2–10.1)
**WOMEN**	***N*** ** = 3,733**	***N*** ** = 3,161**	*N* = 572	*N* = 266	*N* = 306
Mean ± SD	29.2 ± 6.09	29.6 ± 5.92	26.5 ± 6.62 ^**a**^	25.2 ± 6.00 ^**b**^	27.5 ± 6.89 ^**b, c**^
	**min-max**	**%, CI**	**%, CI**	**%, CI**	**%, CI**
1st tercile	2.0–26.0	78.9 (76.7–81.0)	21.1 (19.0-23.3)	10.9 (9.4–12.7)	10.2 (8.7–11.9)
2nd tercile	26.5–31.0	87.7 (86.0-89.3)	12.3 (10.7–14.0)	5.2 (4.2–6.4)	7.1 (5.8–8.4)
3rd tercile	31.5–57.5	92.8 (91.5–94.0)	7.2 (6.0-8.5)	2.0 (1.4–2.8)	5.2 (4.2–6.3)

CVD: Cardiovascular Diseases, SD: Standard Deviation, CI: Confidence interval

^a^*p* < 0.001 as compared to alive (T-test),

^b^*p* < 0.001 as compared to alive; (ANOVA test; *p* values have been adjusted by the Bonferroni correction for multiple comparisons)

*men and women in Health, Alcohol and Psychosocial Factors in Eastern Europe study, Kaunas sample

Table 1 shows GS means and terciles by vital status of men and women in relation to vital status at study follow-up. The mean GS was significantly higher among survivors’ (in separate men and women groups) compared to individuals deceased from all causes of death, from CVD, and deceased from other than CVD causes. In survivor men and women groups, GS minimal values in all terciles were higher as compared to all the three deceased groups.

As shown in Table 3, in both men and women groups, the lowest GS (1st tercile) was associated with a significantly higher risk of all-cause and CVD mortality as compared to the highest levels of GS (3rd tercile) in three Cox regression models: Model 0 (not adjusted), Model 1 (adjusted for age), and Model 2 (adjusted for age and sociodemographic factors). These associations remained statistically significant after full adjustment (in Model 3 adjustment was applied using additionally to variables in Model 1 and 2 such factors as psychological factors, lifestyle factors, and biological factors) for risk of all-cause mortality (HR 1.34 and 1.35 for men and women, respectively) but were not significant for CVD mortality. We observed similar results for the association between decreasing GS and risk of CVD mortality in men but in women such significant associations have been observed only in cases when Models 0, 1, and 2 were applied.

**Table 3:** Association of grip strength with all-cause and cardiovascular mortality in HAPIEE study

Grip strength		All-cause	mortality			CVD	mortality	
categories	Model 0	Model 1	Model 2	Model 3	Model 0	Model 1	Model 2	Model 3
	HR (95% CI)	HR (95% CI)	HR (95% CI)	HR (95% CI)	HR (95% CI)	HR (95% CI)	HR (95% CI)	HR (95% CI)
**MEN**								
Tercile 1	**2.96 (2.50–3.51)**	**1.83 (1.52–2.20)**	**1.53 (1.27–1.85)**	**1.34 (1.10–1.65)**	**3.09 (2.41–3.96)**	**1.75 (1.34–2.29)**	**1.43 (1.09–1.89)**	1.26 (0.93–1.69)
Tercile 2	**1.60 (1.33–1.93)**	**1.21 (1.00-1.46)**	1.15 (0.95–1.39)	1.02 (0.83–1.26)	**1.58 (1.20–2.08)**	1.12 (0.85–1.49)	1.07 (0.80–1.43)	1.00 (0.74–1.36)
Tercile 3 (Ref.)	1.00	1.00	1.00	1.00	1.00	1.00	1.00	1.00
Per 1 kg decrement	**1.06 (1.05–1.06)**	**1.04 (1.03–1.04)**	**1.02 (1.01–1.03)**	**1.02 (1.01–1.03)**	**1.06 (1.05–1.07)**	**1.04 (1.03–1.05)**	**1.02 (1.01–1.04)**	**1.02 (1.004–1.03)**
Per 1 SD decrement	**1.60 (2.26–3.54)**	**1.35 (1.25–1.45)**	**1.22 (1.13–1.31)**	**1.17 (1.08–1.28)**	**1.68 (1.53–1.84)**	**1.38 (1.24–1.53)**	**1.22 (1.10–1.36)**	**1.17 (1.03–1.32)**
**WOMEN**								
Tercile 1	**2.83 (2.50–3.51)**	**1.57 (1.24–1.98)**	**1.33 (1.05–1.69)**	**1.35 (1.03–1.77)**	**5.07 (3.44–7.45)**	**2.19 (1.47–3.26)**	**1.82 (1.21–2.72)**	1.48 (0.95–2.30)
Tercile 2	**1.60 (1.33–1.93)**	1.14 (0.89–1.46)	1.08 (0.84–1.38)	1.18 (0.90–1.56)	**2.50 (1.65–3.78)**	**1.51 (1.47–2.29)**	1.41 (0.92–2.15)	1.32 (0.84–2.08)
Tercile 3 (Ref.)	1.00	1.00	1.00	1.00	1.00	1.00	1.00	1.00
Per 1 kg decrement	**1.07 (1.05–1.08)**	**1.04 (1.02–1.05)**	**1.02 (1.01–1.04)**	**1.02 (1.002–1.04)**	**1.09 (1.07–1.11)**	**1.05 (1.03–1.07)**	**1.03 (1.01–1.06)**	1.02 (0.99–1.04)
Per 1 SD decrement	**1.49 (1.38–1.61)**	**1.24 (1.14–1.35)**	**1.13 (1.04–1.24)**	**1.12 (1.01–1.24)**	**1.71 (1.53–1.90)**	**1.35 (1.19–1.53)**	**1.23 (1.08–1.40)**	1.11 (0.95–1.29)

CI: Confidence interval. CVD: Cardiovascular Diseases. HAPIEE: Health, Alcohol and Psychosocial Factors in Eastern Europe, Kaunas sample; HR: Hazard Ratio. Ref.: Reference. SD: Standard Deviation. Model 0: not adjusted. Model 1: adjusted for baseline age. Model 2: adjusted for all variables in Model 1 plus sociodemographic factors (education, marital status, social activity, and employment status). Model 3: adjusted for all variables in Model 2 plus psychological variables (depressive symptoms, cognitive functions, psychological well-being, and self-rated quality of life), lifestyle variables (smoking habits, alcohol consumption frequency, and physical activity), biologic variables (systolic blood pressure, total cholesterol, triglycerides, fasting glucose, and body mass index), and existing illness at baseline (diabetes, ischemic heart disease, and stroke). Bold typeface indicates significance. Standard deviation (SD) of grip strength: 8.67 for men and 6.09 for women

## Discussion

In the context of the Lithuanian middle-aged and older population, this study is the first to demonstrate once again the association between GS and mortality, as in other populations. The middle-aged and elderly population of urban Lithuanian persons is interesting in that it is a cohort of individuals born after the Second World War, which was followed by difficult living conditions and poverty, and this may have influenced the formation of the musculoskeletal system. In addition, the morbidity and mortality from diseases of the circulatory system in Lithuania remained quite high during the last 4 decades and was one of the highest among North and East European countries. This study assessed the associations of levels of GS with all-cause and CVD mortality in men and women in a prospective, population-based sample during a follow-up period of 12 years. Our results indicate that in survivor groups (men and women) minimal values in all GS terciles were higher as compared to the deceased groups. For both, men and women, the lower GS showed strong associations with a higher risk of all-cause mortality. Decreasing GS was associated with the risk of CVD mortality in men and showed differences in associations in women.

### GS and overall health

At the initial of the current study significant differences of the mean GS in men (48.1 kg) and in women (29.1 kg) have been observed. These differences could have been expected, as GS is a measure of body structure, related to age, sex, and body mass, as well as composition, population, and health [33–36]. The findings from the study defining GS normative reference values for men and women [37] residing in the US obtained mean GS measurements in the age group of 60–64 years 38.4 kg for men and 23.6 kg for women. In a study performed by Ryan McGrath et al. [38] in persons aged at least 50 years, men with GS < 26 kg and women with GS < 16 kg were classified as weak, while men with GS > 32 kg and women with GS > 20 kg were considered strong. Huemer Marie-Theres et al. [39] calculated cut-off points for low GS of 29 kg for men and 18 kg for women. Our investigation was designed to evaluate CVD and all-cause mortality during the period of follow-up in older citizens, who were grouped into terciles of GS, where the weakest, first tercile, included men with 37.7 kg, and women with 21.9 kg in the age groups of 61.8 ± 7.88 and 61.2 ± 7.62 years, respectively.

Kim SH et al. [40] have demonstrated that both, men, and women with low GS (< 28 kg for men and < 18 kg for women) had significantly higher odds of having the lowest level of overall physical fitness. Recent meta-analyses also confirmed the associations between physical activity levels with GS, higher physical activity, and lower sedentary behavior being associated with greater skeletal muscle strength and muscle power [41]. The results of our study revealed that in women the mean of physical activity in leisure time was higher in the highest tercile of GS as compared to the lowest (*p* < 0.05). This study’s results showed significantly higher proportions of married individuals, individuals with university education, high level of social activity, and psychological well-being, normal cognitive function, and proportion of self-rated quality of life as “good” in the 3rd tercile of GS in both groups of men and women. The findings of a significantly lower proportion of disabled individuals, individuals with depressive symptoms, the prevalence of IHD, previous stroke, and individuals who rated their quality of life as “poor” and “very poor” in the highest tercile of GS as compared to the lowest could be indicative of a better level of general health status in both men and women with higher GS measurements. Means of alcohol consumption were significantly higher in the 3rd tercile of GS as compared to the 1st tercile in both groups of men and women which indicates the need for further investigations.

### Risks associated with lower GS

The results from our study are in line with those from the earlier research [12, 17, 42–44] indicative of associations of lower GS with higher all-cause and CVD mortality risk. In a prospective population-based study, with the age range of participants 40–69 years, muscle weakness (defined as GS < 26 kg for men and < 16 kg for women) was associated with a higher hazard for all health outcomes, except colon cancer in women and prostate cancer and lung cancer in both men and women [45]. In our study, the associations of the lowest GS (1st tercile) demonstrated stable results in all the 3 Models applied for the risk of all-cause mortality while adding psychological factors, lifestyle factors, and biological factors did not demonstrate significant associations in the risk for CVD mortality in women. These findings are supported by the evidence coming from a systematic review and meta-analysis [46] where higher levels of GS were significantly associated with a reduced risk of all-cause mortality compared to lower muscular strength, though the opposite to our findings, a slightly stronger association in women than men has been observed. They are supported by the evidence from a PURE study which showed heterogeneity in muscle strength in people living in different countries and country-income settings, and the reduced muscle strength, as measured by GS, has been associated with an increased risk of mortality [16]. The results from a European country survey indicated that an increase of 5 kg in GS was associated with a reduced risk of all-cause, overall cardiovascular mortality; up to a threshold of 42 kg in men and 25 kg in women, increases in GS reduce the risk of all-cause mortality [47]. The findings coming from a population-based study in a sample aged 50 to 75 years from Switzerland [48] concluded though, that GS was associated neither with overall mortality nor with incident cardiovascular events when adjusting for absolute cardiovascular risk.

Our findings indicate that the risk of all-cause mortality and CVD mortality significantly increased when GS was treated as a decrement per 1 kg and decrement per 1 SD. They reflect the study findings, where GS decrease has been associated with all causes and CVD mortality: GS was inversely associated with all-cause mortality (HR per 5 kg reduction in GS 1.16, 95% CI 1.13–1.20; *p* < 0.0001), CVD mortality (HR = 1.17, 95% CI 1.11–1.24; *p* < 0.0001), non-CVD mortality (HR = 1.17, 95% CI 1.12–1.21; *p* < 0.0001), as well as with myocardial infarction and stroke [16], in women and men, respectively, HR per 5 kg lower GS were higher (all at *p* < 0.05) for all-cause mortality and cause-specific mortality from CVD [45]. In our study differences between the results for men and women in prediction of risk of death models for all-cause and CVD mortality have been observed.

In studies, GS has been included in common frailty markers, as well as in frailty phenotype markers for evaluation in the elderly [49, 50], and serum proteomic characteristics of frailty in older adults have been linked with GS as well [51]. According to Nùñez-Lisboa M [52] changes in strength appear to precede changes associated with skeletal muscle morphology, and a change in fibre type from the fast myosin isoform to the slow isoform, which has a lower capacity to generate force has been observed, that could, in turn, contribute to decreased maximal strength in age-related changes. Although phenotypical causes mean GS is lower in women than men, musculoskeletal disorders are more common in women and hand-intensive work leads to an increased risk of these disorders. Knowledge of the gender influence in the rating of work exposure is lacking.

Research in the causes of multifactorial age-related changes in muscle strength, worsening of the function of the neuromuscular system, as well as studies of biomarkers for monitoring the development and progression of frailty in older adults, both in men and women, could be beneficial in adding evidence for better care and prevention of sarcopenia and functional decline in older people without and with chronic conditions.

### Strengths and limitations

Strengths of the study: a prospective design, a large sample size, and a wide age interval of study participants (45–72 years at baseline); a long period of follow-up (from 2006 to 2008 to 2020); standardized and validated study methods were used in our study data collection; adjustments for many potential confounders have been performed; many covariates have been included into the models of statistical analysis.

Recent scientific publications show contrasting results on the association between GS, lower limb muscle strength and physical function [53, 54]. However, we cannot evaluate such associations because, despite many variables measured in our study and included in Cox models, lower limb muscle strength was not measured.

We must admit some other this study limitations: the observational nature of the study does not allow to make strong conclusions on the causal role of GS in all-cause and CVD mortality; the possibility of a recall bias, as lifestyle behaviours were self-reported by the study participants, could lead to the overestimation or underestimation of the determined outcomes; only traditional lifestyle behaviour factors were included into the study; during the period of follow-up no chronic conditions (e.g., oncological, systemic chronic diseases, etc.) have been evaluated, thus providing no evidence their possible impact on the GS results after 12 years. Some observations in differences between the results for men and women need further investigation.

The study population consisted of a random sample from an urban population of Kaunas citizens, thus the generalization of our findings for the Lithuanian population should be done with caution.

## Conclusions

The mean GS was significantly higher among survivors’ men and women compared to individuals deceased from CVD, all causes of death, and deceased from other than CVD causes. The risk of all-cause mortality significantly increased with a decrease in GS. The study adds evidence to the prognostic value of GS and supports the implementation of the measurement of GS into routine healthcare practice, as well as in communities of the elderly population.

## Data Availability

The datasets used and/or analysed during the current study are available from the corresponding author on reasonable request.

## Abbreviations

BMI: body mass index
CHD: coronary heart disease
CI: confidence interval
CVD: cardiovascular disease
DBP: Diastolic Blood Pressure
FG: fasting glucose
GS: grip strength
HAPIEE: Health, Alcohol and Psychosocial Factors in Eastern Europe
HR: hazard ratio
MI: myocardial infarction
PWB: psychological well-being
SD: Standard Deviation
SBP: Systolic Blood Pressure
Tg: triglyceride
